# The Strike of Mine Rescuers

**Published:** 1920-05-01

**Authors:** G. Arbour Stephens

**Affiliations:** Hon. Medical Officer, Swansea Collieries Rescue Station.


### THE STRIKE OF MINE RESCUERS.

**By G. ARBOUR STEPHENS, M.D., B.S., B.Sc. (Lond.), Hon. Medical Officer, Swansea Collieries Rescue Station.**

This is apparently the last thing in strikes. The one feature common to all strikes is that the men come out for more money, never for greater opportunities to become more efficient, and in rescue work efficiency is everything. Efficiency must be considered from the physical as well as the mental point of view, for whilst the men require to have the capacity to be educated or trained for the work, they must also be in a good and sound condition of bodily health in order to stand the strain. Aptitude for being trained demands as one of its essentials a desire to undertake the work, which includes a course of training as well as liability to be called out for very hard and hazardous undertakings.

The usual manner of training the men is to have them down in teams from the collieries in the district; the directors contribute their quota towards the expenses. Some directors, however, take no interest whatever in any work that makes for the men's welfare. Such want of consideration on the part of some of the employers, and the stupid thoughtlessness of others, naturally irritate the men.

Thoughtless Charity.

On the other hand, much of the thoughtless charity that has been indulged in during past years may be held responsible for a great deal of the social disquietude of the present day.

As honorary medical officer of an important Rescue Station, I have had the opportunity of examining a number of the men attending the classes. The practical part of the training consists in carrying out laborious exercises in a specially made gallery filled with noxious fumes, and in order to do this they have to use the oxygen apparatus specially designed for this class of work. My attention was chiefly directed to the condition of the men's hearts. The men are not selected, but, on the contrary, elect to offer themselves—not because they are strong and able-bodied, but for other and varying reasons. For rescue work it is very essential that the men should be quite healthy, as in a poisonous atmosphere their own lives as well as those of the imperilled men are at stake.

Colliers as a Class.

Colliers as a class are credited with being very healthy, which statement seems borne out by the low death-rate amongst them, and one would expect to get a healthy lot of trainées. My experience does not bear this out. At present there is no age limit, so that any man can attend, and, as a consequence, men of all ages present themselves. I suggest that it be laid down as a rule that no man over forty should be accepted. I have seen candidates for training as old as sixty-three, sometimes men with thickened arteries and diseased hearts. Many others had bad teeth, often with pyorrhœa. A septic focus of this sort is obviously dangerous, for we are all agreed that the so-called "soldier's heart" is closely associated with some form of poisoning; hence, for rescue work, it is very essential that the men should be free from any toxin-producing centre. In this connection one might mention the subject of tobacco, which used in excess, as it often is, produces very definite toxic results in a large number of cases. One of the most striking results of smoking I found present in thirteen out of fourteen Irish youths who had come over from Ireland to work in the Swansea area. They all smoked "twist," and they all had markedly toxic hearts. From a labour point of view their worth was low, for their "puff" was impaired.

Volunteers and Pressed Men.

It is said that one volunteer is worth a dozen pressed men, but whilst the spirit may be willing the flesh is ofttimes weak. Why the work should attract the defective more than the healthy is a psychological problem that one cannot work out in this paper. If the men are to be paid, then they must be carefully selected. Of the men whom I examined and who gave the best results were three who had seen very active service—one in Gallipoli, one in Mesopotamia, and one in France.

